# Identification of Downregulated Exosome-Associated Gene ENPP1 as a Novel Lipid Metabolism and Immune-Associated Biomarker for Hepatocellular Carcinoma

**DOI:** 10.1155/2022/4834791

**Published:** 2022-09-26

**Authors:** Zhilan. Li, Qingchun. He, Jinwu. Peng, Yuanliang. Yan, Chencheng. Fu

**Affiliations:** ^1^Department of Pathology, Xiangya Changde Hospital, Changde, China; ^2^Department of Emergency, Xiangya Hospital, Central South University, Changsha, China; ^3^Department of Emergency, Xiangya Changde Hospital, Changde, China; ^4^National Clinical Research Center for Geriatric Disorders, Xiangya Hospital, Central South University, Changsha, China; ^5^Department of Pathology, Xiangya Hospital, Central South University, Changsha, China; ^6^Department of Pharmacy, Xiangya Hospital, Central South University, Changsha, China

## Abstract

Exosome plays an important role in the occurrence and development of tumors, such as hepatocellular carcinoma (LIHC). However, the functions and mechanisms of exosome-associated molecules in LIHC are still underexplored. Here, we investigated the role of the exosome-related gene ENPP1 in LIHC. Comprehensive bioinformatics from multiple databases revealed that ENPP1 was significantly downregulated in LIHC tissues. The patients with downregulated ENPP1 displayed a poor prognosis. Immunohistochemistry (IHC) was used to further confirm the downregulated ENPP1 in LIHC tissues. In addition, the coexpression network of ENPP1 was also explored to understand its roles in the underlying signaling pathways, including fatty acid degradation and the PPAR signaling pathway. Simultaneously, GSEA analysis indicated the potential roles of ENPP1 in the lipid metabolism-associated signaling pathways in the pathogenesis of LIHC, including fatty acid metabolism, fatty acid synthesis, and so on. Finally, immunological analysis indicated that ENPP1 might also be involved in multiple immune-related features, including immunoinhibitors, immunostimulators, and chemokines. Taken together, these findings could enhance our understanding of ENPP1 in LIHC pathogenesis and immune response and provide a new target for ENPP1-related immunotherapy in clinical treatment.

## 1. Introduction

The 5-year survival rate of hepatocellular carcinoma (LIHC) ranks second among all cancers [1, 2]. In terms of diagnosis, as the only serum biomarker widely used in daily practice, alpha-fetoprotein (AFP) has low sensitivity and specificity in the early diagnosis of LIHC [3]. In terms of treatment, immunotherapy has been applied to LIHC; however, the response of patients to immunotherapy is still limited [4, 5, 6]. Therefore, it is crucial to improve our understanding of the complex pathogenesis of LIHC.

Exosomes are a subset of extracellular vesicles (EVs) with a diameter of 40–160 nm [7, 8]. Exosomes contain a variety of substances, such as proteins, amino acids, nucleic acids, lipids, and metabolites, and can mediate cell-to-cell communication [9, 10]. Furthermore, exosomes have been shown to play an important role in the regulation of the immune system [11, 12]. Recent reports have demonstrated the potential roles of exosomes in the tumorigenesis and progression of cancers [13], including LIHC [14]. However, the detailed molecular mechanisms of exosome-associated genes in LIHC have not been fully elucidated.

Ectonucleotide pyrophosphatase/phosphodiesterase 1 (ENPP1), also known as plasma cell glycoprotein 1 (PC-1), is a type II transmembrane glycoprotein with nucleotide pyrophosphatase and phosphodiesterase activities [15]. Nikonorova et al. demonstrated the biological function of exosome-loaded ENPP1 in mediating intercellular communication, involving various physiological and pathological states [16]. In recent years, ENPP1 has been found to play an important role in immune responses to various stimuli [17]. Studies have shown that aberrantly expressed ENPP1 participates in the pathogenesis and therapeutic response of human cancers, including ovarian cancer, glioma, and breast cancer [18, 19, 20]. However, the diagnostic value and functional mechanism of ENPP1 in LIHC have not been explored.

Here, we comprehensively evaluated the expression profiles and potential prognostic values of ENPP1 in LIHC. We demonstrated that the exosome-related gene ENPP1 was significantly downregulated in LIHC. Moreover, the patients with downregulated expression of ENPP1 showed a poor prognosis. Immunological analysis revealed the association between ENPP1 levels and immune infiltrating cells in LIHC. Taken together, these data collectively suggested that ENPP1 could be a promising biomarker for LIHC prognosis and immune response and may serve as a new immunotherapy-associated target.

## 2. Materials and Methods

### 2.1. Data Acquisition and Bioinformatics Analysis

Three public LIHC datasets, GSE6764 [21], GSE14323 [22], and GSE14520 [23], were downloaded from the Gene Expression Omnibus (GEO) database. Then, the differently expressed genes (DEGs) between the normal liver tissues and LIHC were screened with the following criteria: *P* value <0.01 and |logFC| > 1 (Table 1). Next, Venn plots were employed to identify the co-DEGs among the exosome-associated gene dataset (Supplementary Table 1) [24] and the above-mentioned GEO datasets. After then, Xiantao Xueshu [25], TNMplot [26], and UALCAN [27] were used to confirm the downregulated expression levels of ENPP1 in LIHC tissues.

The prognostic values of co-DEGs in LIHC patients were explored by the Kaplan–Meier plotter [28]. The prognostic indexes mainly included disease-specific survival (DSS) and overall survival (OS). Subsequently, the LinkedOmics platform [29] was used to analyze the coexpressed molecules associated with ENPP1. At the same time, using LinkedOmics, we performed the enrichment analysis of ENPP1 coexpressed molecules, including gene ontology (GO) and Kyoto encyclopedia of genes and genomes (KEGG). Using single-sample GSEA (ssGSEA) in Xiantao Xueshu and TISIDB [30], we explored the roles of ENPP1 in the immune-associated features in LIHC patients.

### 2.2. Immunohistochemistry (IHC)

The paraffin-embedded LIHC samples and corresponding peritumoral samples were obtained from Xiangya Hospital, Central South University. The ethics was approved by Xiangya Hospital, Central South University (No. 202205113). Immunohistochemistry (IHC) was performed using a universal two-step IHC staining kit (PV-9000, ZSGB-BIO, Beijing, China) according to the instructions. The primary antibody used in this study was anti-ENPP1 (1 : 500, ab223268, Abcam). The IHC results were identified according to the staining percentage and staining intensity.

### 2.3. Statistical Analysis

On the Kaplan–Meier platform, the comparison of OS and DSS between tumor and normal groups was performed using the log-rank test. A Cox risk proportional regression model was used to analyze and calculate hazard ratios (HRs). The Mann–Whitney *U* test was used for comparison of normal and tumor specimens, and the Wilcoxon test was used for comparison of tissues with its matched adjacent specimens. All the critical values of statistical significance were *P* < 0.05.

## 3. Results

### 3.1. Identification of the DEGs between the LIHC Group and the Normal Group

The DEGs between LIHC and normal liver tissue were analyzed from three GEO datasets, GSE6764, GSE14323, and GSE14520. We identified 830 upregulated and 866 downregulated molecules in GSE6764, 505 upregulated and 590 downregulated molecules in GSE14520, and 343 upregulated and 258 downregulated molecules in GSE14323 (Supplementary Table 2).

**Figure 1:**
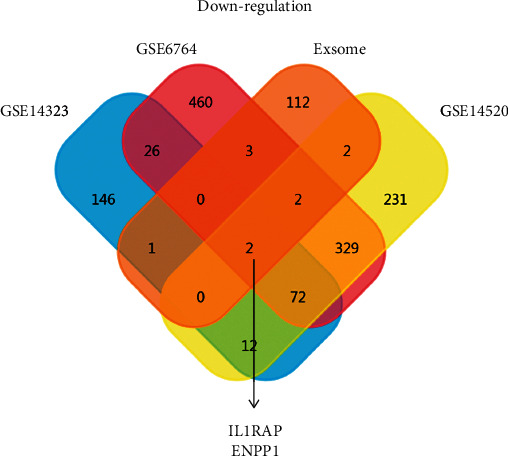
Identification of co-DEGs between the exosome-associated gene dataset and three GEO-LIHC datasets. In this Venn plot, we found two co-downregulated molecules, IL1RAP, and ENPP1, in LIHC tissues.

In order to explore the role of exosome-associated genes in LIHC, we used Venn analysis to screen the co-DEGs between the three GEO datasets and the exosome-associated gene dataset. As shown in Figure 1, we identified two codownregulated molecules, interleukin 1 receptor accessory protein (IL1RAP) and ENPP1 in LIHC tissues. However, no coupregulated molecules were found in Supplementary Figure 1.

### 3.2. The Prognosis Values of IL1RAP and ENPP1 in LIHC Patients

**Figure 2:**
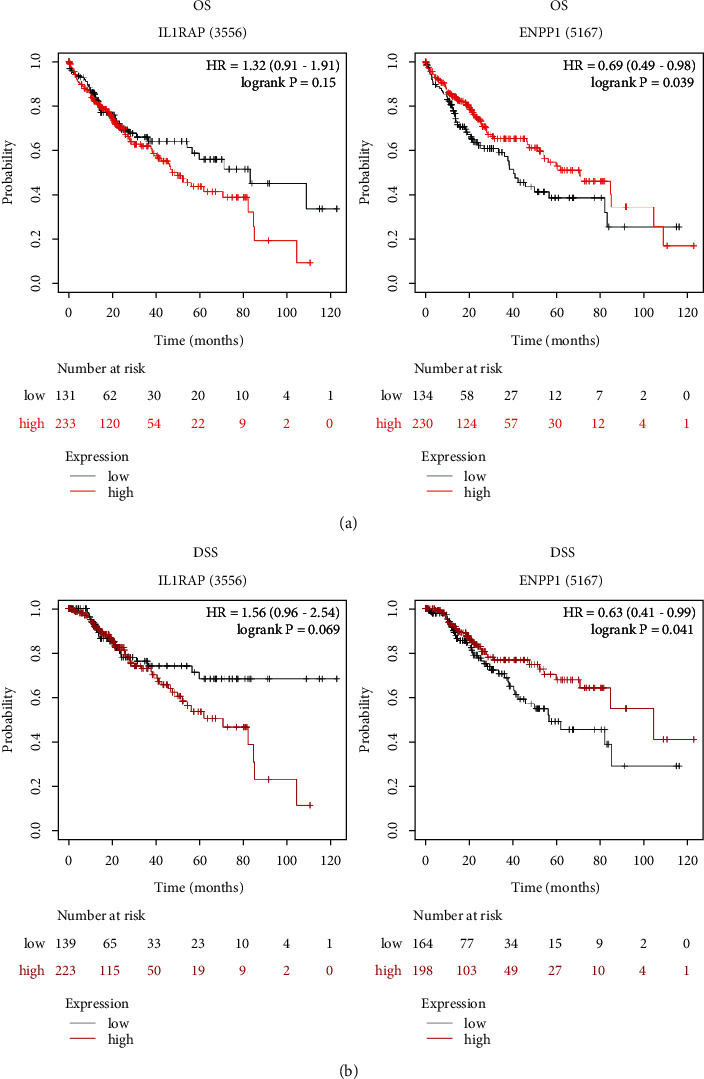
The effect of IL1RAP and ENPP1 on the patients' prognosis in LIHC. (a-b) The Kaplan–Meier plotter database indicated the prognostic values of aberrantly expressed IL1RAP and ENPP1 in LIHC patients, including overall survival and disease-specific survival.

To explore whether aberrant expression of IL1RAP and ENPP1 affected the patients' prognosis in LIHC, we used the Kaplan–Meier plotter database and found that patients with a high ENPP1 level displayed a good OS rate (hazard ratio (HR) = 0.69, 95% CI = 0.49–0.98, *P*=0.039) and DSS (HR = 0.63, 95% CI = 0.41–0.99, *P*=0.041), whereas, there was no clear connection between the level of IL1RAP and LIHC patients' prognosis (Figures 2(a)-2(b)). Therefore, these data collectively revealed the important prognostic roles of ENPP1 expression in LIHC.

### 3.3. ENPP1 Was Confirmed to Be Downregulated in LIHC

**Figure 3:**
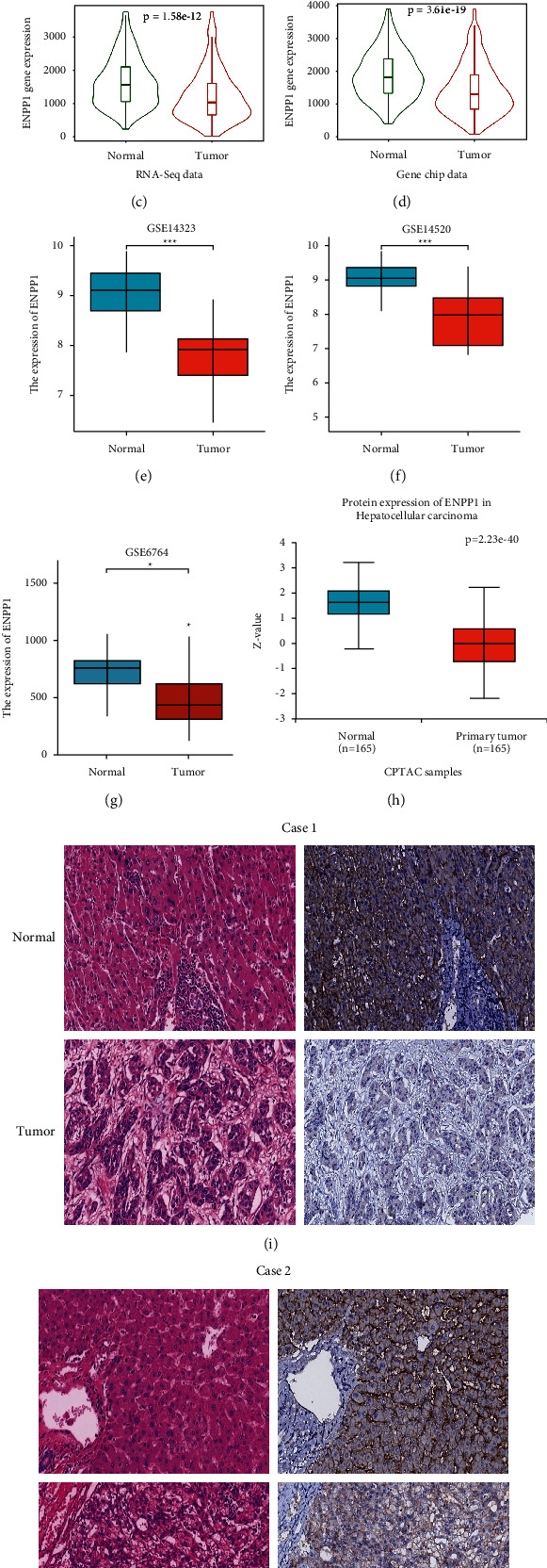
Downregulation of ENPP1 in LIHC patients. (a-b) Xiantao Xueshu indicated the downregulated expression level of ENPP1 in TCGA-LIHC, (c-d) TNMplot database depicted the downregulated ENPP1 mRNA in LIHC tissues. (e-g) ENPP1 expression was significantly diminished in the three GEO-LIHC datasets. (h) UALCAN indicated the downregulated protein expression of ENPP1 in LIHC tissues, and (i-j) IHC results confirmed that ENPP1 was significantly downregulated in tumor tissues compared with adjacent tissues (^*∗*^*P* < 0.05, ^*∗∗*^*P* < 0.01, and ^*∗∗∗*^*P* < 0.001).

To investigate the role of ENPP1 in LIHC progression, the TCGA-LIHC dataset in Xiantao Xueshu was used to predict ENPP1 mRNA expression patterns in 374 liver cancer samples and 50 normal tissue samples. As shown in Figure 3(a), the results showed that the expression level of ENPP1 mRNA in liver cancer tissues was lower than that in normal liver tissues. In addition, the expression of ENPP1 was confirmed to be significantly downregulated in 50 LIHC specimens compared with matched adjacent samples (Figure 3(b)). Next, the RNA-seq data and gene chip data in TNMplot showed that the expression of ENPP1 mRNA in cancer tissues was lower than that in normal liver tissues (Figures 3(c)-3(d)). We also confirmed that the expression of ENPP1 was significantly downregulated in tumor groups from the three abovementioned GEO datasets, GSE6764, GSE14323, and GSE14520 (Figures 3(e)–3(g)). In addition, the UALCAN database was used to demonstrate the downregulated protein expression level of ENPP1 in LIHC (Figure 3(h)), indicating that the protein expression and mRNA expression of ENPP1 in different databases were consistent. Accordingly, our IHC data also showed that ENPP1 expression was significantly downregulated in tumor tissues compared with paracancerous tissues (Figures 3(i)-3(j)). These results collectively suggested that ENPP1 may play an inhibitory role in the occurrence and development of LIHC.

### 3.4. The Enrichment of ENPP1 Coexpression Network in LIHC

**Figure 4:**
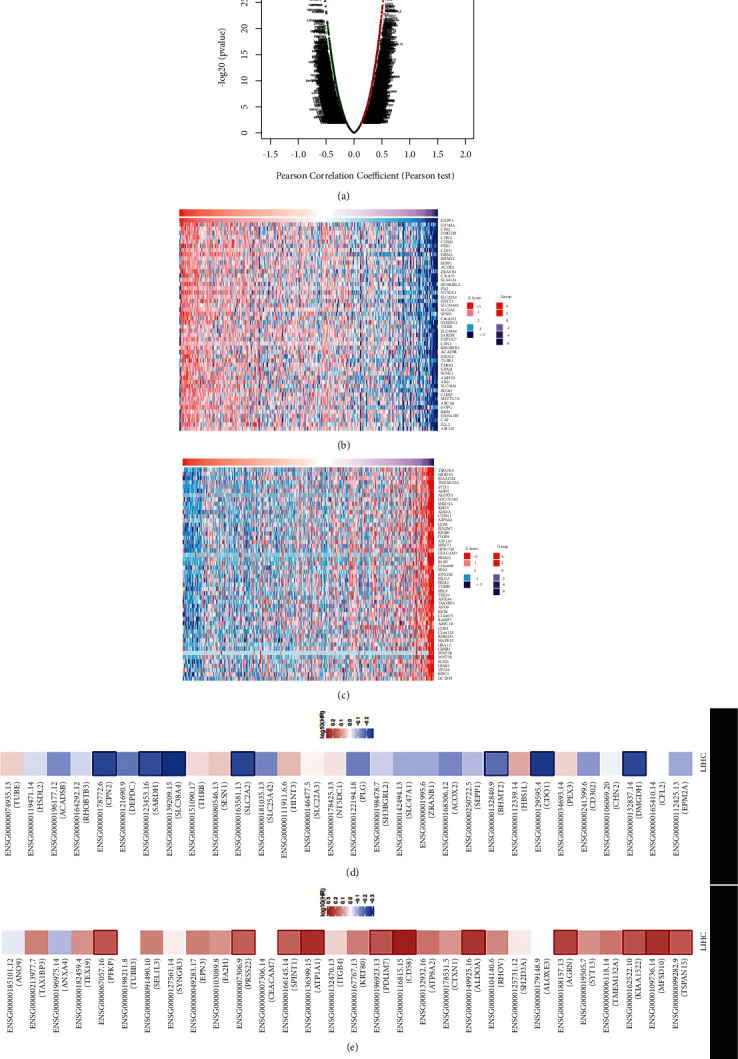
The ENPP1 coexpression molecules in LIHC from LinkedOmics. (a) The volcano plots showed the coexpressed molecules correlated with ENPP1, (b-c) the heatmap showed the top 30 coexpressed molecules correlated with ENPP1 in LIHC, and (d-e) the prognostic values of the coexpressed molecules were correlated with ENPP1 in LIHC.

To explore the underlying biological significance of ENPP1 in LIHC, we analyzed the coexpression pattern of ENPP1 in TCGA-LIHC through LinkedOmics. The volcano plots showed that the coexpressed molecules were positively (red dots) and negatively (blue dots) correlated with ENPP1 (Figure 4(a)). The heatmap displayed the top 30 molecules that were positively and negatively correlated with ENPP1 in LIHC (Figures 4(b)-4(c), Supplementary Table 3, Supplementary Table 4). Interestingly, the top 30 positively-associated molecules might be the low-risk biomarkers for LIHC patients, with 13/30 molecules possessing the protective HR (Figure 4(d)). Conversely, the top 30 negatively-associated molecules might be the high-risk biomarkers for LIHC patients, with 14/30 negative molecules possessing an unfavorable HR (Figure 4(e)).

**Figure 5:**
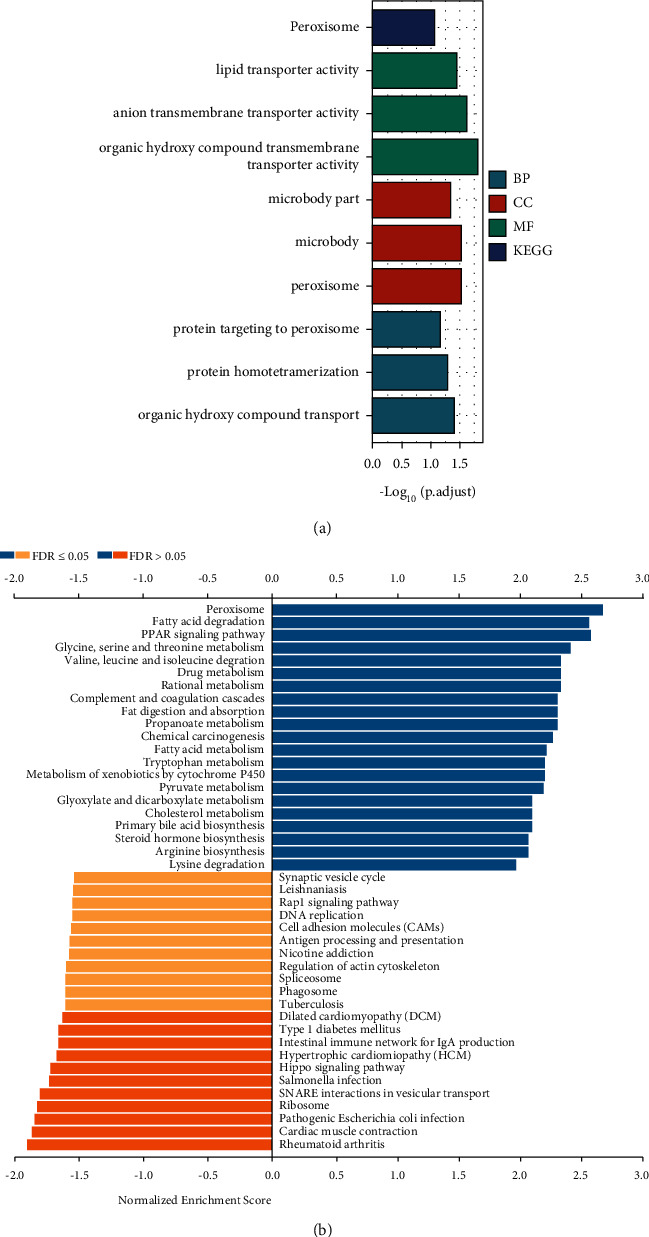
The functional enrichment of ENPP1 coexpressed molecules in LIHC. (a) GO enrichment of ENPP1 coexpressed molecules in LIHC and (b) the KEGG signaling pathway of ENPP1 coexpressed molecules in LIHC.

**Figure 6:**
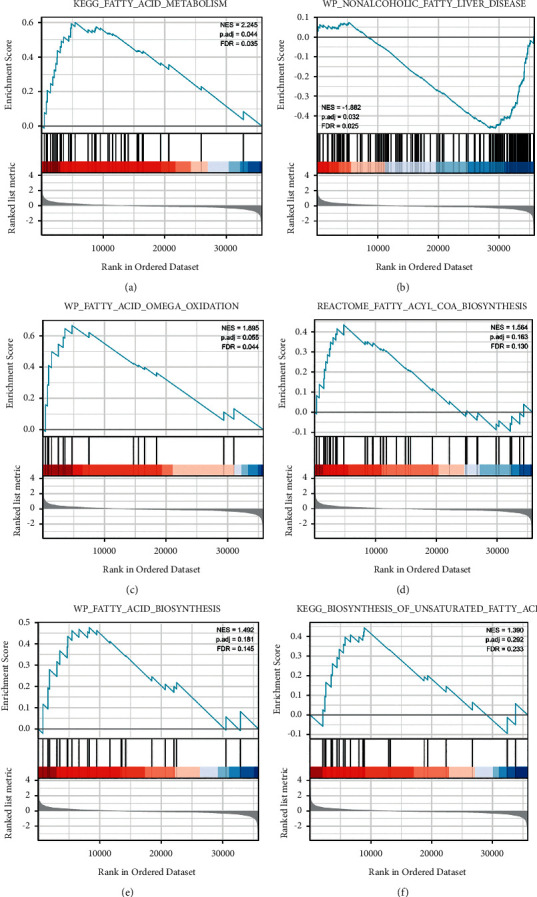
The GSEA enrichment analysis of ENPP1 differentially expressed genes in LIHC. (a-f) The fatty acid metabolism, nonalcoholic fatty liver disease, fatty acids omega oxidation, fatty acyl COA biosynthesis, fatty acid biosynthesis, and the biosynthesis of unsaturated fatty acid pathways were enriched in ENPP1 differentially expressed genes in LIHC.

Moreover, GO enrichment analysis conveyed that ENPP1 coexpressed molecules mainly participated in the regulation of several biological processes (BP), such as organic hydroxy compound transmembrane transporter activity, anion transmembrane transporter activity, and lipid transporter activity. As for the cellular components (CC), ENPP1 coexpressed molecules mainly took part in the regulation of the microbody and peroxisome. As for the molecular function (MF), the coexpressed genes of ENPP1 were significantly involved in the regulation of organic hydroxy compound transport (Figure 5(a)). Moreover, the KEGG enrichment analysis conveyed that the enriched signaling pathways of ENPP1 coexpressed molecules were fatty acid degradation, PPAR signaling pathway, and others (Figure 5(b)). At the same time, GSEA analysis was performed to identify several fatty metabolic pathways that could be significantly regulated by ENPP1-associated molecules, such as fatty acid metabolism and nonalcoholic fatty liver disease (Figures 6(a)-6(b)). We also found that ENPP1 might participate in the regulation of other fatty metabolic pathways, such as fatty acid omega oxidation and fatty acid biosynthesis (Figures 6(c)–6(f)).

### 3.5. The Regulatory Roles of ENPP1 in Immune Regulation

**Figure 7:**
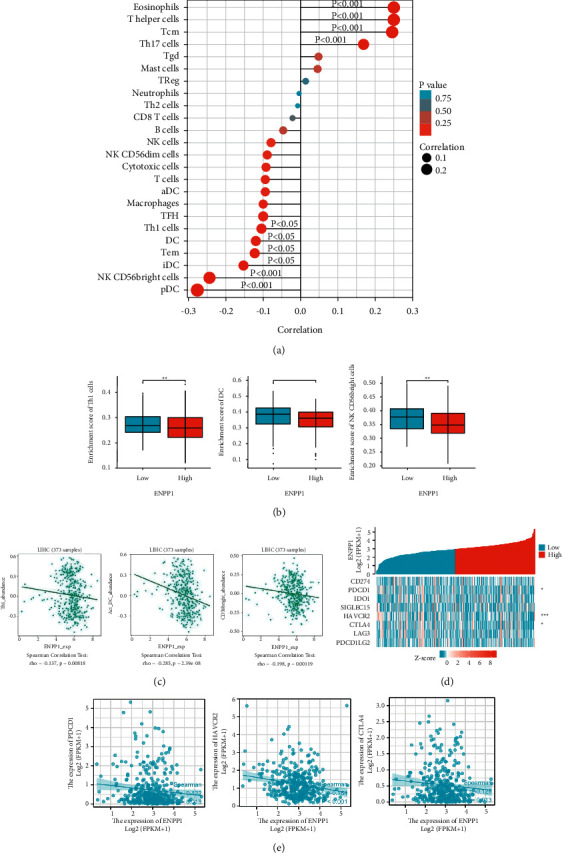
Relationship between ENPP1 expression and tumor-infiltrating immune cells in LIHC. (a) The Xiantao Xueshu indicated the correlation between ENPP1 level and the infiltrating immune cells, (b) the histogram showed the downregulated Th1 cells, DC, and NK CD56bright in ENPP1-highly expressed group, and (c) the histogram shows the negative relationship between ENPP1 and infiltration of Th1 cells, DC, and NK CD56bright. (d) Heat map and (e) scatter plot showed the negative correlation between ENPP9 and three immune checkpoints, PDCD1, HAVCR2, and CTLA4.

To assess whether ENPP1 expression levels were related to the tumor-infiltrating immune cells in LIHC, the ssGSEA algorithm in Xiantao Xueshu was used to show the association between ENPP1 expression and several immune infiltrating cells, such as dendritic cells (DCs), CD56 (bright) natural killer cell (NK CD56bright), and Th1 cells (Figure 7(a)). Similarly, the immune infiltrating cells, such as Th1 cells, DC, and NK CD56bright, were significantly downregulated in the ENPP1-highly expressed group (Figure 7(b)). Furthermore, the negative associations between ENPP1 expression and infiltration of Th1 cells, DC, and NK CD56bright were confirmed by the TISIDB platform (Figure 7(c)).

Next, we explored whether ENPP1 levels were associated with the immune checkpoints in LIHC. The heatmap (Figure 7(d)) and scatterplot (Figure 7(e)) showed a negative correlation between ENPP1 expression and three immune checkpoints, which include programmed cell death protein 1 (PDCD1), hepatitis A virus cellular receptor 2 (HAVCR2), and cytotoxic T-lymphocyte associated protein 4 (CTLA4).

**Figure 8:**
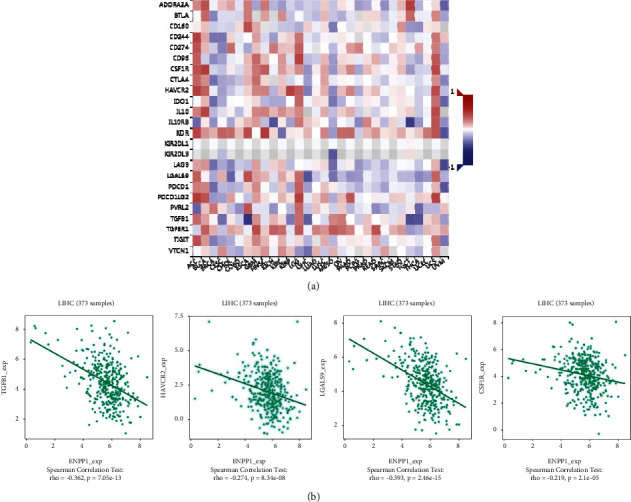
The relationship between ENPP1 and immunoinhibitors in TCGA-LIHC patients. (a) The heatmap indicated the association between ENPP1 and several immunoinhibitors. (b) The correlation analysis between several immunoinhibitors, TGFB1, HAVCR2, LGALS9, and CSF1R, and ENPP1 expression.

**Figure 9:**
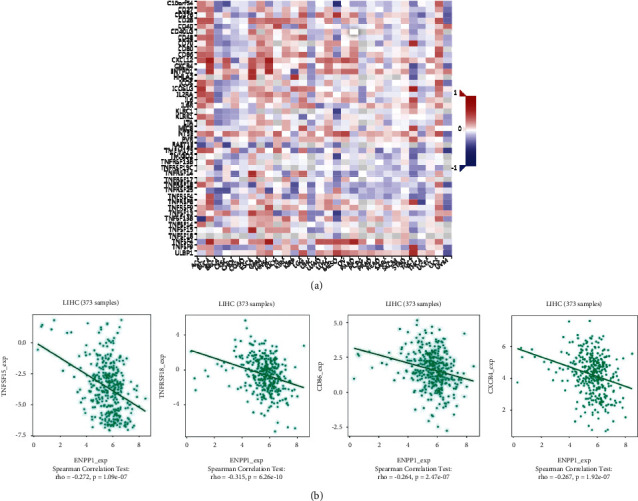
The relationship between ENPP1 and immunostimulators in TCGA-LIHC patients. (a) The heatmap indicated the association between ENPP1 and several immunostimulators. (b) The correlation analysis between several immunostimulators, TNFSF15, TNFRSF18, CD86, and CXCR4, and ENPP1 expression.

We used the TISIDB platform to explore the underlying roles of ENPP1 in several immune-associated signatures, including immunoinhibitors and immunostimulators.  Figure 8(a) demonstrates the association between ENPP1 expression and several immunoinhibitors in TCGA-LIHC patients. The results showed that ENPP1 was significantly negatively correlated with the following immunoinhibitors, TGFB1 (Spearman *r* = −0.362, *P*=7.05*e* − 13, *P*=7.05*e* − 13), HAVCR2 (Spearman *r* = −0.274, *P*=8.34*e* − 08), LGALS9 (Spearman *r* = −0.34e−08), −0.393, *P*=2.46*e* − 15), and CSF1R (Spearman *r* = −0.219, *P*=2.1*e* − 05) (Figure 8(b)).  Figure 9(a) demonstrates the association between ENPP1 expression and several immunostimulators in TCHA-LIHC patients. The results showed that ENPP1 was significantly negatively correlated with the following immunostimulators, TNFSF15 (Spearman *r* = −0.272, *P*=1.09*e* − 07), TNFRSF18 (Spearman *r* = −0.315, *P*=6.26*e* − 10), CD86 (Spearman *r* = −0.264, *P*=2.47*e* − 07), and CXCR4 (Spearman *r* = −0.267, *P*=1.92*e* − 07) (Figure 9(b)). We further explored the relationship between ENPP1 and chemokines and chemokine receptors. Supplementary Figure 2A shows the relationship between ENPP1 expression and chemokines in TCGA-LIHC patients. The chemokines were negatively-correlated with ENPP1 and mainly included CXCL1 (Spearman *r* = −0.334, *P*=4.82*e* − 11), CCL26 (Spearman *r* = −0.342, *P*=1.48*e* − 11), CXCL8 (Spearman *r* = −0.336, *P*=3.43*e* − 11), and CXCL3 (Spearman *r* = −0.289, *P*=1.45*e* − 08) (Supplementary Figure 2B). Supplementary Figure 3A shows the association between ENPP1 expression and chemokine receptors. The chemokine receptors were negatively-correlated with ENPP1 and mainly included CCR5 (Spearman *r* = −0.195, *P*=0.00016), CCR10 (Spearman *r* = −0.135, *P*=0.00916), CXCR3 (Spearman *r* = −0.207, *P*=5.64*e* − 05), and CXCR4 (Spearman *r* = −0.267, *P*=1.92*e* − 07) (Supplementary Figure 3B). Taken together, these data suggested the promising roles of aberrant ENPP1 in the regulation of multiple immune-related signals in LIHC.

## 4. Discussion

The tumor microenvironment (TME) is an important intrinsic link in the occurrence, development, invasion, and metastasis of LIHC. Exosomes are increasingly recognized as professional information carriers in TME regulation [31, 32], which play important roles in tumor therapeutic response [33]. A growing number of studies have shown that exosomes can affect LIHC progression from multiple aspects, such as angiogenesis, chemoresistance, and immune response. Dai et al. found that downregulation of exosomal CLEC3B in LIHC promotes cell metastasis and angiogenesis through AMPK and VEGF signaling [34]. Cho et al. found that exosomal microRNA-4661-5p could be used as a potential diagnostic biomarker for early LIHC [35]. Circulating exo-miR-1307-5p has been shown to promote cell metastasis in LIHC [36]. The above results suggest that exosomes play a crucial role in LIHC development, and an in-depth exploration of their mechanisms may help to discover new therapeutic strategies. However, the detailed roles of exosome-related gene ENPP1 in LIHC have not been reported. Using comprehensive bioinformatics platforms, we would like to investigate the underlying roles of exosome-related molecules in LIHC in this report. By exploring the co-DEGs between the exosome-associated dataset and three GEO-LIHC datasets, we found that the exosome-associated molecule ENPP1 was significantly downregulated in LIHC patients and was correlated with unfavorable patient prognosis. LinkedOmics also indicated the roles of ENPP1 coexpressed genes in the prognosis of LIHC patients.

Emerging studies have shown aberrant ENPP1 in cancer pathology. Hu et al. demonstrated that dysregulated ENPP1 increases the malignancy of human lung cancer by inducing epithelial-mesenchymal transition and stem cell characteristics [37]. Wang et al. demonstrated that high expression of ENPP1 in high-grade serous ovarian cancer predicts a poor prognosis and therapeutic response [18]. These studies have demonstrated that ENPP1 plays an important role in the development and treatment of tumors. In our study, ENPP1 was downregulated in LICH tissues compared with normal liver tissues. The LIHC patients with high ENPP1 expression had a good prognosis.

The immune microenvironment is formed by complex interactions between tumor cells and the host immune response [38]. LIHC shows a high degree of malignant biological properties, which is closely related to the suppression of host immune response [31]. NK cells play a very important role in the prevention of LIHC and have been considered a potential cell therapy resource. NK cell dysfunction is involved in multiple mechanisms leading to the occurrence of LIHC [4]. Additionally, in LIHC, regulatory DCs produce indoleamine-2,3-dioxygenase (IDO) to promote tumor immune escape [39]. Studies have shown that infiltration of Th17 cells correlate with poor prognosis in LIHC [40]. These results indicate that NK cells, DCs, and Th17 are closely related to LIHC. In this paper, the roles of ENPP1 in the regulation of the immune environment were studied. The results showed that ENPP1 was significantly negatively correlated with NK CD56bright cells, DC cells, and Th1 cells. These results suggested that ENPP1 might be a promising biomarker for immunotherapy in LIHC patients.

## 5. Conclusions

In conclusion, we demonstrated that exosome-associated ENPP1 was downregulated in LIHC and correlated with patient prognosis. In addition, ENPP1 might be involved in the occurrence and development of hepatocellular carcinoma by affecting the immune cell infiltration. Therefore, our study revealed that ENPP1 might be a promising biomarker for LIHC.

## Figures and Tables

**Table 1:** The features of three GEO datasets on gene expression.

GEO datasets	Platform	Sample size		DEGs	References
		Cancer	Normal		
GSE6764	GPL570	35	10	854 upregulated genes and 893 downregulated genes	[21]
GSE14323	GPL571	38	19	343 upregulated genes and 258 downregulated genes	[22]
GSE14520	GPL571	22	21	632 upregulated genes and 650 downregulated genes	[23]

## Data Availability

The data used to support the findings of this study are included within the supplementary information files.

## Supplementary Materials

Supplementary Materials Supplementary Figure 1. Identification of coupregulated molecules between the exosome-associated gene dataset and three GEO-LIHC datasets. Supplementary Figure 2. The relationship between ENPP1 and chemokines in TCGA-LIHC patients. (a) The heatmap indicated the association between ENPP1 and several chemokines and (b) the correlation analysis between several chemokines, CXCL1, CCL26, CXCL8, and CXCL3, and ENPP1 expression. Supplementary Figure 3. The relationship between ENPP1 and chemokine receptors in TCGA-LIHC patients. (a) The heatmap indicated the association between ENPP1 and several chemokine receptors and (b) the correlation analysis between several chemokine receptors, CCR5, CCR10, CXCR3, and CXCR4, and ENPP1 expression. Supplementary Table 1. The exosome-associated gene dataset. Supplementary Table 2. The DEGs between LIHC and normal liver tissue were analyzed from three GEO datasets, GSE6764, GSE14323, and GSE14520. Supplementary Table 3. The top 30 genes were positively linked with ENPP1 in LIHC. Supplementary Table 4. The top 30 genes were negatively linked with ENPP1 in LIHC.
