# Supplementary figures and images for: Identification of Downregulated Exosome-Associated Gene ENPP1 as a Novel Lipid Metabolism and Immune-Associated Biomarker for Hepatocellular Carcinoma
Source: J Oncol. 2022 Sep 26;2022:4834791. doi: 10.1155/2022/4834791 (PMC9529392; doi:10.1155/2022/4834791)

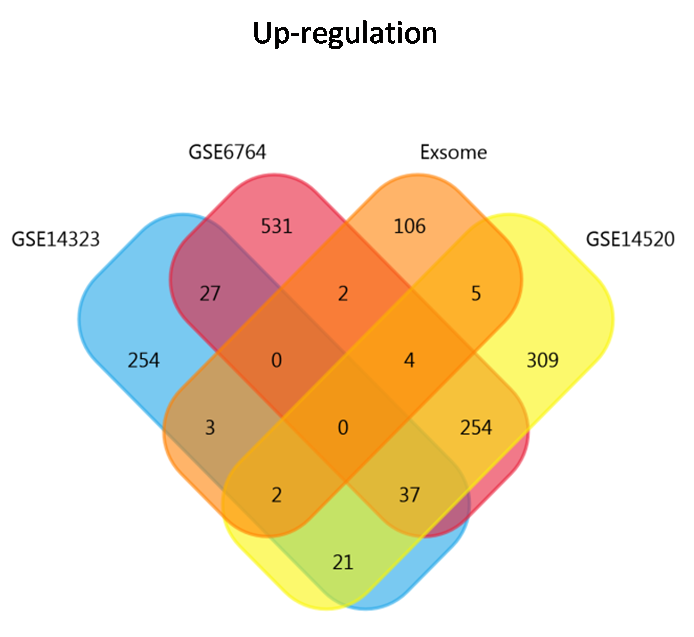

Supplement: Supplementary Materials — Supplementary Figure 1. Identification of coupregulated molecules between the exosome-associated gene dataset and three GEO-LIHC datasets. Supplementary Figure 2. The relationship between ENPP1 and chemokines in TCGA-LIHC patients. (a) The heatmap indicated the association between ENPP1 and several chemokines and (b) the correlation analysis between several chemokines, CXCL1, CCL26, CXCL8, and CXCL3, and ENPP1 expression. Supplementary Figure 3. The relationship between ENPP1 and chemokine receptors in TCGA-LIHC patients. (a) The heatmap indicated the association between ENPP1 and several chemokine receptors and (b) the correlation analysis between several chemokine receptors, CCR5, CCR10, CXCR3, and CXCR4, and ENPP1 expression. Supplementary Table 1. The exosome-associated gene dataset. Supplementary Table 2. The DEGs between LIHC and normal liver tissue were analyzed from three GEO datasets, GSE6764, GSE14323, and GSE14520. Supplementary Table 3. The top 30 genes were positively linked with ENPP1 in LIHC. Supplementary Table 4. The top 30 genes were negatively linked with ENPP1 in LIHC. [file 4834791.f1.zip › Supplementary Figure 1.png]

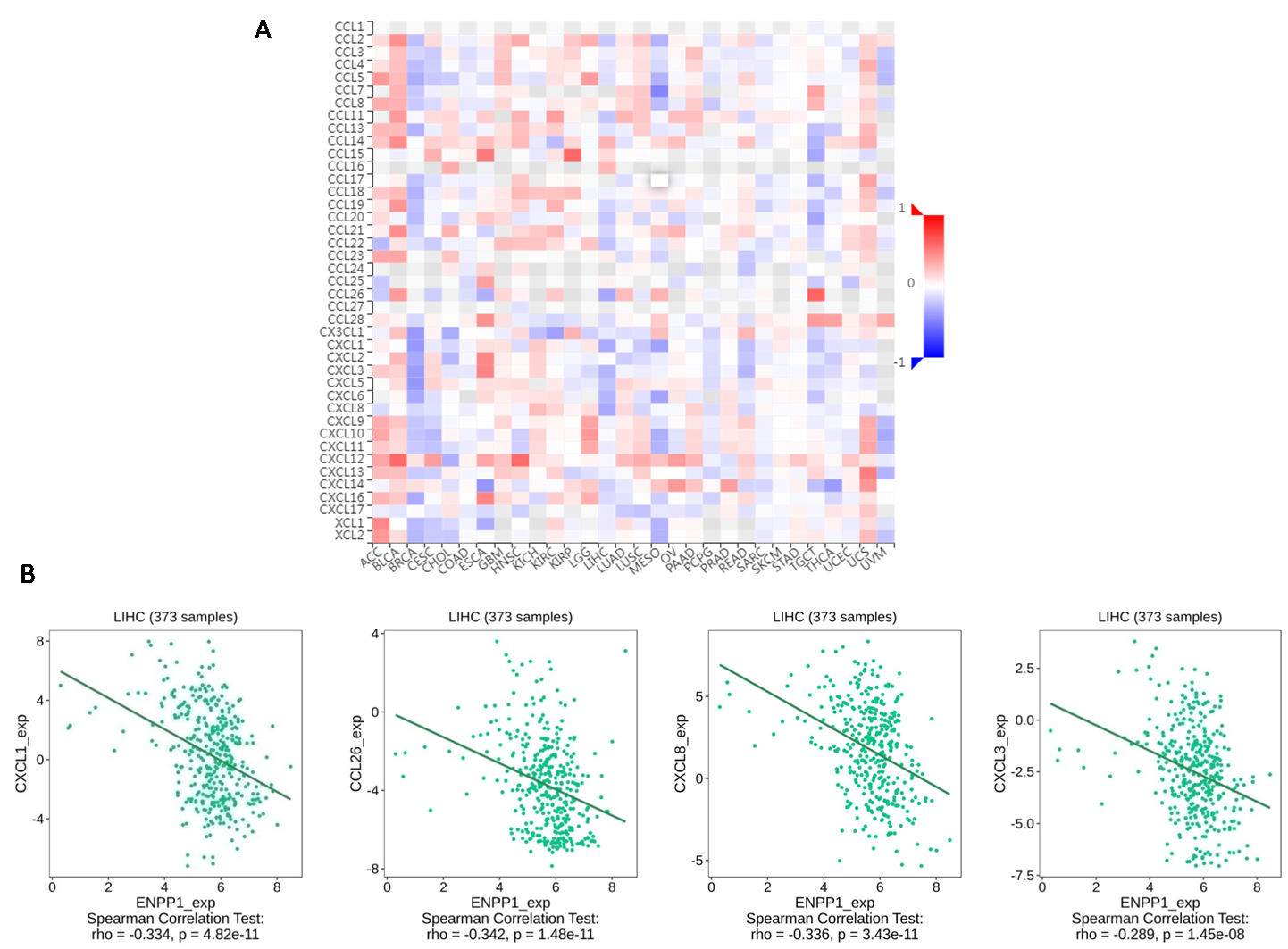

Supplement: Supplementary Materials — Supplementary Figure 1. Identification of coupregulated molecules between the exosome-associated gene dataset and three GEO-LIHC datasets. Supplementary Figure 2. The relationship between ENPP1 and chemokines in TCGA-LIHC patients. (a) The heatmap indicated the association between ENPP1 and several chemokines and (b) the correlation analysis between several chemokines, CXCL1, CCL26, CXCL8, and CXCL3, and ENPP1 expression. Supplementary Figure 3. The relationship between ENPP1 and chemokine receptors in TCGA-LIHC patients. (a) The heatmap indicated the association between ENPP1 and several chemokine receptors and (b) the correlation analysis between several chemokine receptors, CCR5, CCR10, CXCR3, and CXCR4, and ENPP1 expression. Supplementary Table 1. The exosome-associated gene dataset. Supplementary Table 2. The DEGs between LIHC and normal liver tissue were analyzed from three GEO datasets, GSE6764, GSE14323, and GSE14520. Supplementary Table 3. The top 30 genes were positively linked with ENPP1 in LIHC. Supplementary Table 4. The top 30 genes were negatively linked with ENPP1 in LIHC. [file 4834791.f1.zip › Supplementary Figure 2.png]

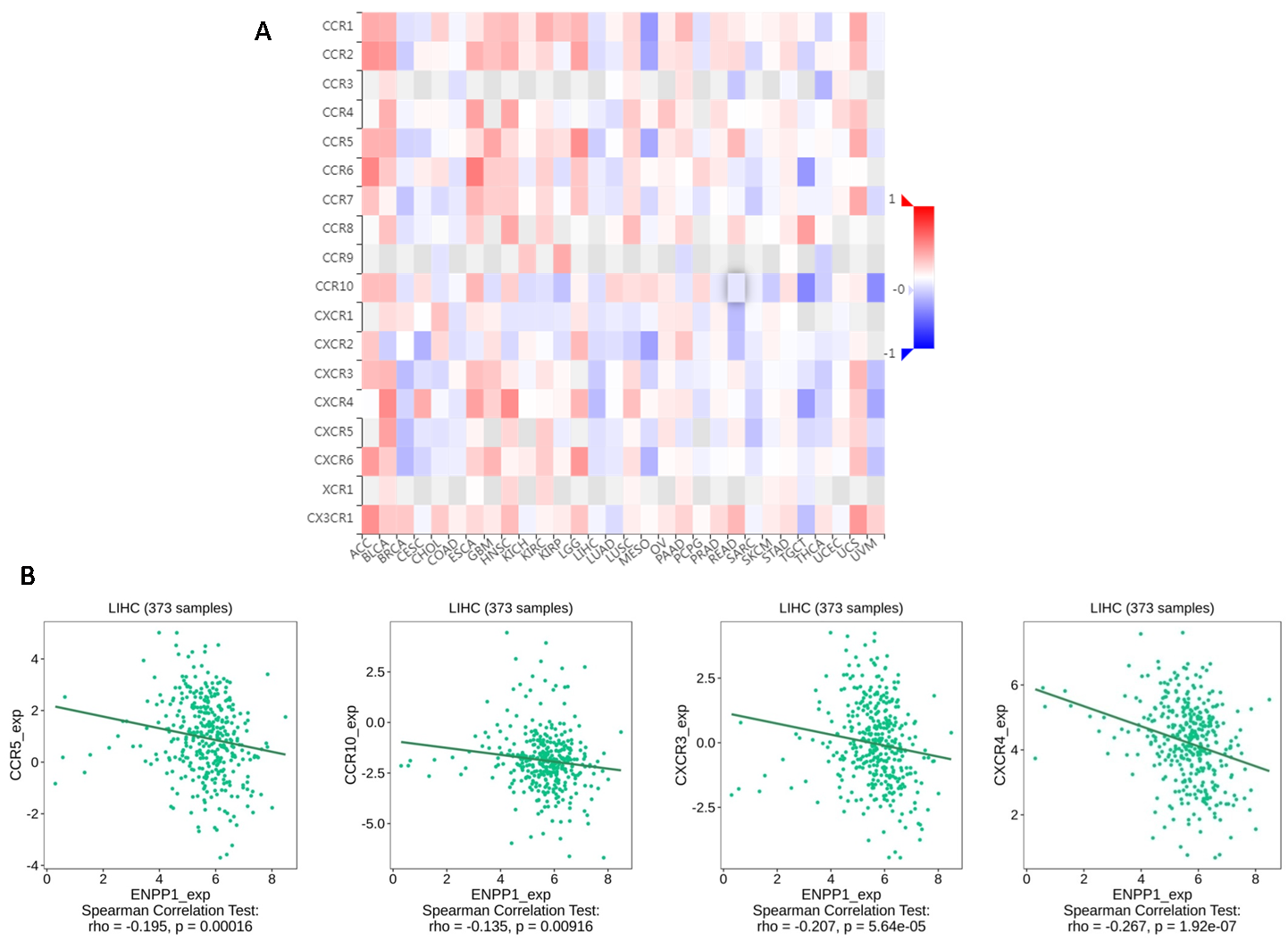

Supplement: Supplementary Materials — Supplementary Figure 1. Identification of coupregulated molecules between the exosome-associated gene dataset and three GEO-LIHC datasets. Supplementary Figure 2. The relationship between ENPP1 and chemokines in TCGA-LIHC patients. (a) The heatmap indicated the association between ENPP1 and several chemokines and (b) the correlation analysis between several chemokines, CXCL1, CCL26, CXCL8, and CXCL3, and ENPP1 expression. Supplementary Figure 3. The relationship between ENPP1 and chemokine receptors in TCGA-LIHC patients. (a) The heatmap indicated the association between ENPP1 and several chemokine receptors and (b) the correlation analysis between several chemokine receptors, CCR5, CCR10, CXCR3, and CXCR4, and ENPP1 expression. Supplementary Table 1. The exosome-associated gene dataset. Supplementary Table 2. The DEGs between LIHC and normal liver tissue were analyzed from three GEO datasets, GSE6764, GSE14323, and GSE14520. Supplementary Table 3. The top 30 genes were positively linked with ENPP1 in LIHC. Supplementary Table 4. The top 30 genes were negatively linked with ENPP1 in LIHC. [file 4834791.f1.zip › Supplementary Figure 3.png]
